# ZAP70: A Key Gene Identified by Differential Expression Analysis for Early Diagnosis of Fetuses with Emanuel Syndrome

**DOI:** 10.1007/s10528-024-10808-3

**Published:** 2024-04-30

**Authors:** Jing Hu, Mengyue Wang, Ruiyao Xiang

**Affiliations:** Department of Obstetrics and Reproductive Medicine Center, The Affiliated Hospital of Yunnan University, Kunming, 650021 Yunnan China

**Keywords:** Emanuel syndrome, t(11;22), Chromosomal translocation, +der[22]t(11;22)(q23;q11)

## Abstract

Emanuel syndrome is a rare autosomal disorder characterized by microcephaly, heart defects, cleft palate and developmental delay. However, there is a lack of specific prenatal screening for Emanuel syndrome. To screen for early diagnostic marker genes in fetuses with karyotype+der[22]t(11;22)(q23;q11) of Emanuel syndrome. Transcriptome sequencing and clinical trait data of t(11;22)(q23;q11) translocation samples were screened from the GEO database. The differentially expressed genes (DEGs) were screened by principal component analysis of gene expression by R package, and intersections were taken with balanced and unbalanced DEGs. Then, the correlation with clinical traits was determined by WGCNA analysis, GO and KEGG enrichment analysis, and then univariate Cox analysis and Lasso analysis were performed to obtain the key genes. The core regulatory genes were obtained after protein–protein interaction (PPI) network analysis. A total of 50 DEGs were obtained after differential analysis. WGCNA analysis showed that DEG was associated with the chromosomal imbalance and age module. GO and KEGG enrichment analyses showed candidate genes were associated with exocytic vesicle membrane, synaptic vesicle membranes, glycoprotein complex, dystrophin-associated glycoprotein complex and malaria. COX and Lasso analyses yielded 5 hub genes, including *ZBED9, RGS20, SGCB, ETV5*, and *ZAP70*. The results of PPI identified the key regulatory gene associated with chromosomal imbalance as the *ZAP70* gene. *ZAP70* may be a key gene for early diagnosis of Emanuel syndrome in fetuses with+der[22]t(11;22)(q23;q11) karyotype.

## Introduction

Emanuel syndrome (ES), also known as supernumerary der[22]t(11;22) syndrome with partial der[22] trisomy(Manju et al. 2022), is a rare inherited chromosomal disorder(Piwowarczyk et al. 2022), usually resulting from a 3:1 meiosis in carriers with the t(11;22)(q23;q11) translocation(Zackai and Emanuel 1980). Patients with this trisomy are characterized by severe developmental delay, pre and postnatal growth deficits, microcephaly, ear abnormalities, preauricular tag or invagination, and genital abnormalities (Li and Zhu 2022; Luo et al. 2017). To date, the majority of ES cases have been diagnosed in the postnatal period and only a few prenatal cases have been reported (Luo et al. 2020). Specific diagnostic methods of ES are still limited to prenatal invasive procedures such as chorionic villus sampling, amniocentesis, or cordocentesis. However, most pregnant women are reluctant to undergo such procedures to detect fetal abnormalities because of concerns about the risks associated with the invasive procedure. Although nonspecific ultrasound indicators such as intrauterine growth retardation, anomalies of the posterior cranial fossa, cardiac malformations, and other abnormalities can be applied for auxiliary diagnosis, amniotic fluid karyotyping is still required to diagnose a fetus with ES (Hao et al. 2022). In addition, only 16% of ES patients reported ultrasound abnormalities (Carter et al. 2009). Some phenotypes are difficult to diagnose by prenatal ultrasound, such as facial malformations, ear and genital anomalies, heart defects, and hypotonia. Moreover, these ultrasound findings do not distinguish fetuses with ES from other fetuses with genetic syndromes. The clinical data from five hospitals in Guangdong Province showed that between January 2015 and July 2021, only six ES fetuses with structural malformations were diagnosed by ultrasound (Luo et al. 2020). As far as methods and techniques for early diagnosis of ES are not yet available, there is an urgent need to improve the efficiency of ES diagnostic methods and reduce the probability of ES neonates. This study aims to investigate the hub gene to help targeted diagnosis for ES.

## Materials and Methods

### Data Sources

The dataset GSE13122 was obtained from GEO (http://www.ncbi.nlm.nih.gov/geo) for analysis of Emanuel syndrome data analysis, including transcriptome data and sample information of chromosome translocation of 9 t(11;22)(q23;q11) translocation (balanced translocation carriers), 4 ES individuals and 13 normal controls.

### Differential Gene Screening

The study used the facto extra package of R software to analyze the expression of the genes by principal component analysis (PCA). Subsequently, the limma package was used to analyze the differences between the translocation samples and the control samples, and the balanced translocation samples and the unbalanced translocation samples, respectively. Both comparisons were screened for differentially expressed genes (DEGs) with *P* < 0.05 and |log2(Foldchange)|> 0.58, and the intersection genes of DEGs in the two groups were used as the candidate differential gene.

### WGCNA Analysis of DEGs

The candidate genes obtained above were analyzed by gene clustering using the R package WGCNA. The power with the squared value of the correlation coefficient that reached 0.8 or more for the first time was selected. Secondly, based on the clustering and dynamic tree cutting method, the number of genes in the module was set to 10, and the gene modules were identified by the dynamic tree cutting method, while modules with the degree of similarity of more than 0.5 were clustered and merged. Finally, by calculating the correlation between modules and clinical phenotypes, the gene set modules closely related to clinical status were identified. Here, we selected gene modules that were significantly related to unbalanced translocation for subsequent analysis. Meanwhile, we analyzed the gender-related gene modules and further performed KEGG and GO enrichment to explore the underlying mechanism.

### Model Construction and Model Reliability Verification

Based on the set of candidate differential genes, the preliminary candidate genes were firstly obtained by single-factor analysis according to whether the translocation were balanced or unbalanced. Then, the preliminary candidate genes were analyzed by Lassoof Lars package to screen out the covariant genes. After that, the bio-marker genes were obtained. Subsequently, the training set was randomly selected at a ratio of 0.7, the glm and RandomForest models were constructed, and the AUC was calculated and the ROC curve was plotted in the remaining samples, so as to verify the reliability of the marker genes.

### PPI Analysis and Functional Enrichment of Key Marker Gene

In order to further clarify the functions involved in marker genes, the STRING database was used to select proteins with experimental validation for the interactive analysis, and 5 and 10 were used as the number of interaction nodes to mine the core regulatory genes. Moreover, the PPI network of key factor-acting proteins was constructed through the STRING database (https://string-db.org/) and performed functional enrichment analysis. Subsequently, we selected the genes with experimentally verified relationships among the interactions and performed GO and KEGG enrichment analyses.

## Results

### Differentially Expressed Gene Analysis

**Fig. 1:**
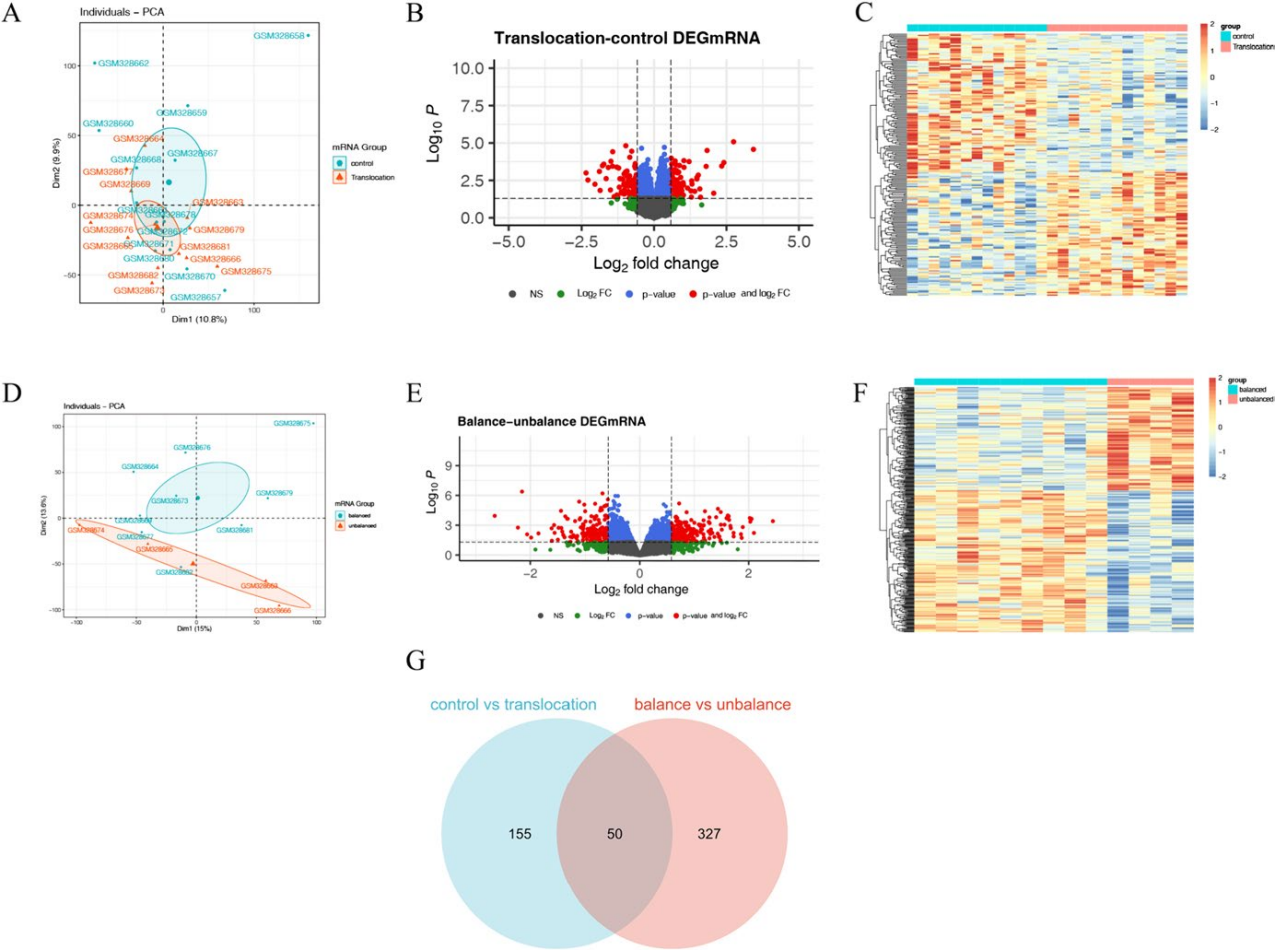
Analysis of differentially expressed genes. **A** PCA analysis of normal vs. translocation samples; **B** Normal vs. translocation differential gene volcano plot; **C** Normal vs. translocation differential gene heatmap; **D** PCA analysis of equilibrium vs. unbalanced translocation samples; **E** Equilibrium vs. unbalanced translocation samples differential gene volcano plot; **F** Equilibrium vs. unbalanced translocation samples differential gene heatmap; **G** Differential gene Weyenne plots

The results of PCA showed difference in overall expression between the translocation group and the normal group in the GSE13122 dataset. The expression difference analysis was filtered by *P* < 0.05 and |log2FC|> 0.58 (Fig. 1A). As a result, a total of 205 DEGs were obtained, of which 108 were significantly highly expressed and 97 were significantly low expressed in the translocation samples (Fig. 1B, C). PCA analysis of balanced translocation chromosomes and unbalanced translocation chromosomes in the samples. The results showed dispersion of samples between groups and clustering of samples within groups. (Fig. 1D). Balance-unbalance differential gene screening was performed with *P* < 0.05 and |log2FC|> 0.58, resulting in a total of 377 DEGs, of which 218 were significantly highly expressed and 159 genes were significantly low expressed (unbalance) (Fig. 1E, F). To obtain t(11; 22)(q23; q11) biomarker genes, DEGs in the translocation-control group were intersected with the balance-unbalance translocation group, and a total of 50 intersected genes were obtained, which were used as the subsequent screening of t(11; 22)(q23; q11) biomarker marker candidate genes (Fig. 1G).

### Key Modules Screening by WGCNA

**Fig. 2:**
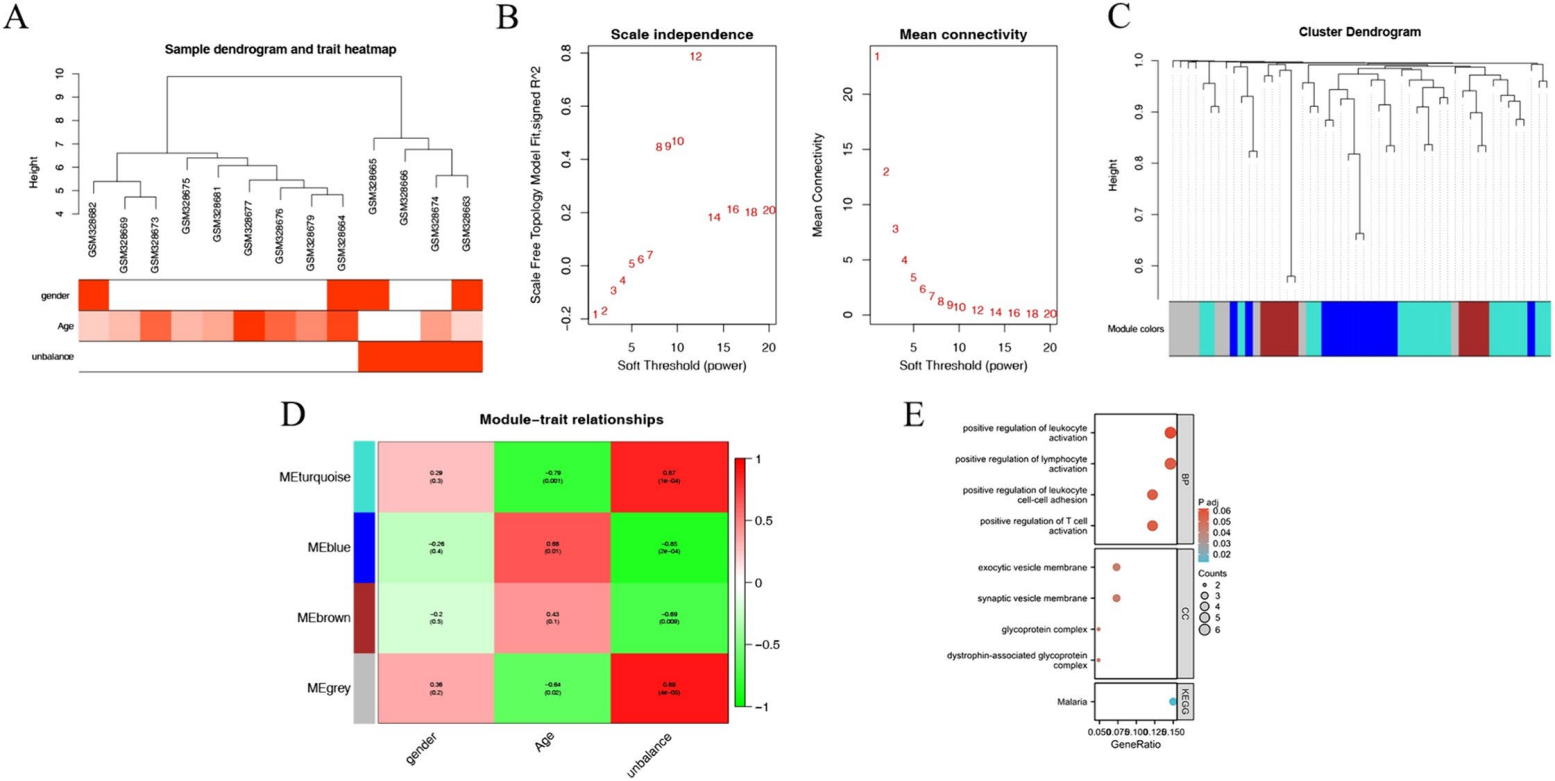
WGCNA analysis of key modules and GO/KEGG functional enrichment analysis of genes. **A** Sample clustering and clinical trait heatmap; **B** Analysis of soft threshold parameters vs. average connectivity; **C** Gene clustering tree vs. gene modules; **D** Module vs. trait heatmap; **E** Candidate gene GO/KEGG functional enrichment bubble map

Fifty candidate biomarker genes were further screened based on WGCNA analysis, and the relationship between the 50 genes and clinical indicators was analyzed simultaneously. The clinical information of the integrated translocation samples included clinical indicators such as gender, age, and disequilibrium, and the expression of the 50 candidate genes in the translocation samples was used for the modular correlation analysis (Fig. 2A). A soft threshold of 12 was determined to enable the adjacent function to better satisfy the scale-free condition and R2 > 0.8 (Fig. 2B). Significant correlations with unbalance were obtained based on the clustering and dynamic tree cutting method for the MEturquoise module (Cor = 0.87, *P* = 1e-04), MEblue (Cor = − 0.85, *P* = 2e-04), and MEbrown (Cor = − 0.69, *P* = 0.009) (Fig. 2C–D). The genes in MEturquoise module (Cor = − 0.79, *P* = 0.001) and MEblue (Cor = 0.68, *P* = 0.01) were correlated with age, suggesting that translocations affecting the expression of certain genes that is correlated with age (Fig. 2D). The results of GO revealed that genes in key modules were enriched on exocytic vesicle membrane, synaptic vesicle membrane, glycoprotein complex, dystrophin-associated glycoprotein complex (GO, Cellular Component, *P* < 0.05) and Malaria (KEGG, *P* < 0.05), suggesting that the key genes related to ES were mainly associated with exocytic vesicle membrane, synaptic vesicle membrane, glycoprotein complex, and dystrophin-associated glycoprotein complex, as well as with malaria (Fig. 2E).

### Prediction Model Construction

**Fig. 3:**
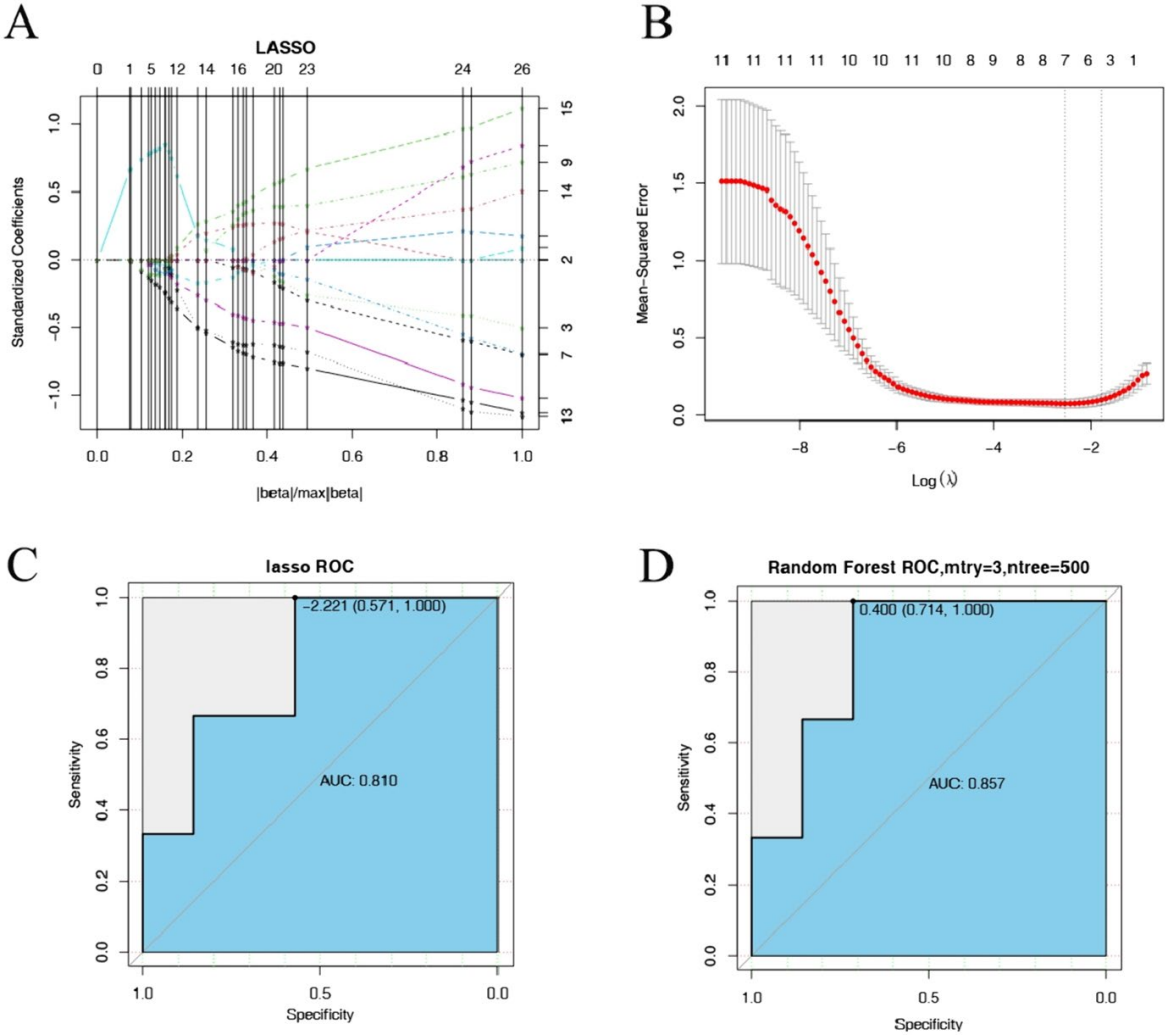
Maker gene prediction model. **A**–**B** Characterized genes screened by the LASSO algorithm; **C** AUC of LASSO screened genes; **D** AUC of RandomForest modeled genes

The 50 candidate genes were analyzed by univariate Cox regression analysis to determine the balanced translocation samples and unbalanced translocation samples, and the results are shown in Table 1 (only 20 genes are shown). 11 candidate genes were obtained with *P* < 0.04 (Lasso regression failed under *P* < 0.05 criterion, probably because of more covariate genes, so the more significant genes were chosen to screen out some covariate genes), and 11 candidate genes were further screened by using the Lasso regression (Fig. 3A). Five marker genes, including *ZBED9, RGS20, SGCB, ETV5*, and *ZAP70*, were finally obtained (Fig. 3B). In order to verify the reliability of the five marker genes in the samples, the GML model and random Forest model were constructed with five marker genes, respectively. In addition, the samples with a proportion of 0.7 were used as the training set, and the samples with a proportion of 0.3 were used for validation, and the AUC were obtained to be 0.810, and 0.857, respectively (Figs. 3C, D), suggesting that the reliability of marker genes was significant.

**Table 1:** Univariate Cox analysis of candidate genes

Gene	Coef	Exp(coef)	Se(coef)	z	Pr( >|z|)
NLRP11	0.997254	2.71082787	0.556256	1.792797	0.073005
IGHD	− 0.67149	0.51094677	0.359938	− 1.86557	0.062101
THAP10	0.928405	2.53046986	0.497327	1.866789	0.061931
DPYSL2	0.4275	1.53341909	0.226966	1.883543	0.059627
ZNF711	0.850521	2.34086499	0.439604	1.934744	0.053022
CLEC2B	− 1.02725	0.3579915	0.526808	− 1.94994	0.051183
VCAM1	0.867611	2.38121457	0.442001	1.962917	0.049656
LYPD6B	0.638498	1.89363473	0.320223	1.993919	0.046161
LPIN1	0.939515	2.55873891	0.46258	2.03103	0.042252
WASF1	− 0.77715	0.45971597	0.380689	− 2.04142	0.041209
SGCB	0.974021	2.64857176	0.466926	2.086027	0.036976
ETV5	1.464594	4.32578804	0.685002	2.138088	0.03251
C14orf182	0.961452	2.61549124	0.445567	2.157818	0.030942
ZBED9	2.17308	8.78529804	1.006942	2.158098	0.03092
NRIP1	− 1.04376	0.35212789	0.475308	− 2.19597	0.028094
MEIS2	1.169133	3.21920076	0.523068	2.235146	0.025408
SLC12A8	0.701393	2.01655947	0.308947	2.270267	0.023191
SCARF1	− 1.25379	0.28542173	0.549579	− 2.28136	0.022527
RGS20	0.63433	1.88575793	0.265721	2.387198	0.016977
TMOD1	1.169186	3.21937228	0.489068	2.390642	0.016819
ZAP70	− 1.02911	0.35732329	0.426683	− 2.4119	0.01587

### PPI Analysis

**Fig. 4:**
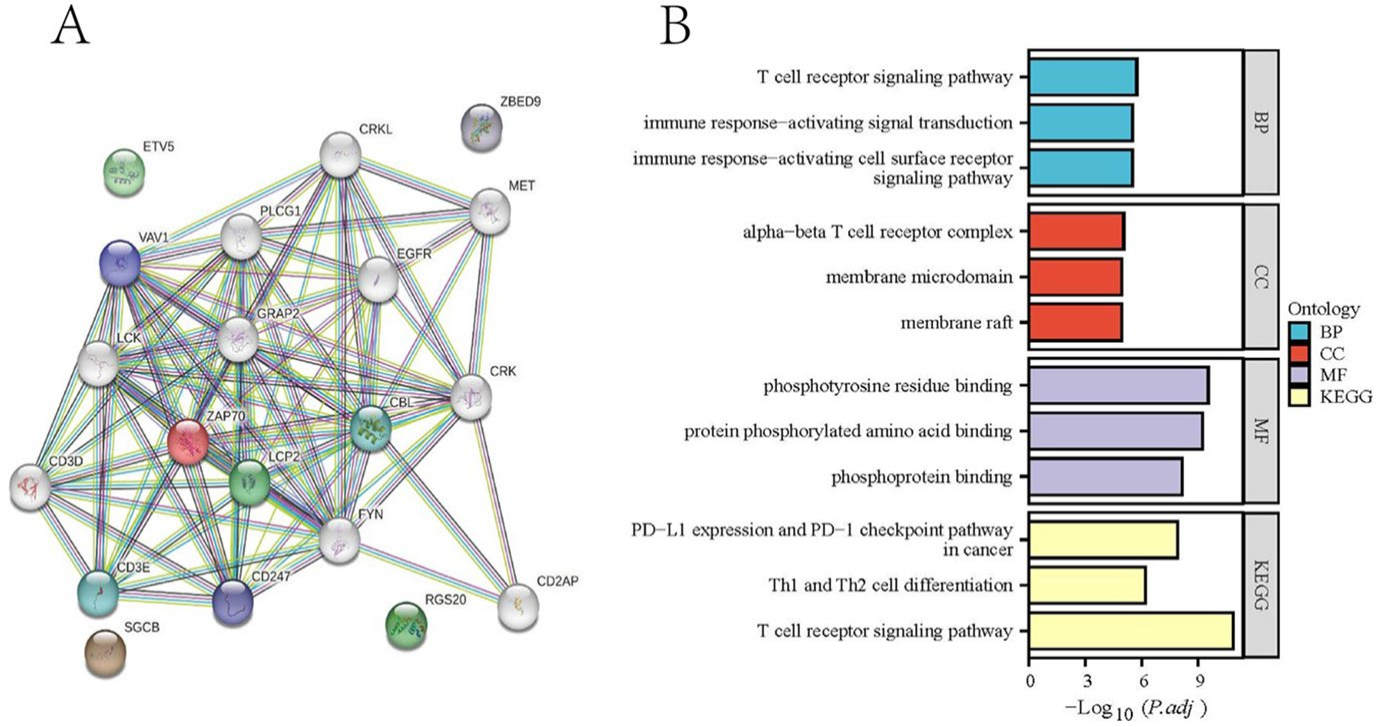
PPI and enrichment analysis of marker genes. **A** PPI network; **B** Enrichment analysis of maker genes and interaction proteins

The results of PPI network and functional enrichment for 5 marker genes showed that *ZAP70* was the key regulatory gene, and those directly interacting with *ZAP70* were *VAV1, CBL, LCP2, CD247*, and *CD3E* (Fig. 4A). Figure 4B showed that these genes were enriched to pathways such as signal transduction, and immune responses activate cell surface receptor signaling pathway. Cellular components were significantly enriched for α-β T-cell receptor complexes, membrane microdomains, membrane rafts, etc., and molecular functions were significantly enriched for phosphotyrosine residue binding, protein phosphorylation amino acid binding, and phosphoprotein binding, etc. KEGG analysis showed that PD-L1 expression. The PD-1 checkpoint pathway and the Th1 and Th2 cell differentiation signaling pathways were significantly enriched in ES.

## Discussions

The t(11;22)(q23;q11) chromosomal balanced translocation is the only recurrent non-Robertsonian translocation in humans (Fu et al. 2018). Carriers generally have a normal phenotype, but male translocation carriers may have infertility, azoospermia, or decreased sperm count and motility (Kara et al. 2014). Female carriers present with a history of recurrent spontaneous abortions and births of malformed children. The main reason for the occurrence of these symptoms is all due to abnormal chromosome segregation, resulting in abnormal chromosome copy number of the embryo (Chen et al. 2022). In translocation carriers, abnormal 3:1 segregation of quadriradial chromosome during meiosis results in the birth of offspring with ES.

ES is a rare chromosomal disorder with a variety of distinctive congenital anomalous phenotypes, such as severe intellectual disability, heart defects, and high arched palate (Ohye et al. 2014). When ES fetuses have no obvious developmental defects, the fetal abnormalities cannot be accurately detected by ultrasound examination and other techniques (Hao et al. 2022). ES patients die in the neonatal period and rarely survive into childhood. The detection of chromosome deletions and duplications remains challenging and places a financial burden on the families of the patients (Hardisty and Vora 2014). To date, because there are no practical, specific, and sensitive diagnostic methods for prenatal screening for ES (Carter et al. 2009; Chen et al. 1996), it is very difficult to know whether a fetus has ES before birth. Although nonspecific ultrasound indicators such as intrauterine growth retardation, anomalies of the posterior cranial fossa, cardiac malformations, and gastrointestinal abnormalities can suggest the diagnosis, some of these children have no obvious anomalies, and therefore cannot be accurately screened for ES (Walfisch et al. 2012).

Although noninvasive prenatal genetic testing (NIPS) detects common autosomal aneuploidies and genetic disorders early in pregnancy by maternal plasma testing (Minear et al. 2015), and 2 ES fetuses were screened by this way in 2020. However, the efficacy of NIPS is uncertain due to low coverage, fetal fractions, and microduplications, and NIPS also cannot be used as a diagnostic method for the specific detection of ES fetuses because of the small chromosomal region of abnormality as well as the double segmental repeats with 11q23 and 22q11 in ES (Luo et al. 2020). Defects of the posterior fossa (62% of fetuses; 13/21) and left diaphragmatic hernia (29% of fetuses; 6/21) are the most frequently reported prenatal findings in ES syndrome. No pattern of specific prenatal ultrasound markers of ES exists (Piwowarczyk et al. 2022). Abnormalities of the posterior fossa are frequent and may be diagnosed as early as in the first trimester of pregnancy. Specific diagnosis can be made only after invasive genetic testing (Walfisch et al. 2012). So it is critically important to screen for the presence of diagnostic markers of ES in early life, which would avoid the negative impacts of having high-frequency miscarriages or delivering malformed fetus who are carriers of the balance translocation.

In this study, 50 DEGs were obtained by transcriptome analysis in the public GEO database and all were associated with imbalance translocation. Five key genes were obtained by univariate Cox regression and Lasso regression analyses, and the PPI network identified *ZAP70* as a key regulatory gene for supernumerary der[22]t(11;22). *ZAP70* is a protein kinase that regulates spindle assembly and chromosome arrangement in oocytes(Kim et al. 2017). In the present study, we found that *ZAP70* was significantly Highly expressed in ES fetuses, so the use of NIPS to detect maternal peripheral blood while specifically detecting the expression of ZAP70. Therefore, the use of NIPS to detect the expression of ZAP70 in maternal peripheral blood specifically at the same time provides a theoretical basis for the noninvasive prenatal detection of ES fetuses. It can also reverse the speculation that the parents who conceived ES fetuses may be t(11;22)(q23;q11) carriers, so as to scientifically guide the carriers to give birth to healthy fetuses and reduce the physical and mental trauma of the carriers. However, this study is based on a small sample size and database analysis, which has not been validated by experiments. In the future, we will further validate the results of this study by using peripheral blood from mothers with confirmed ES fetuses in the clinic, which will provide new perspectives for non-invasive prenatal trisomy detection.

## Data Availability

Data sharing not applicable to this article as no datasets were generated or analyzed during the current study.
